# Anticoccidial and immunogenic effectivity of encapsulated organic acids and anticoccidial drugs in broilers infected with *Eimeria* spp.

**DOI:** 10.1038/s41598-022-20990-2

**Published:** 2022-10-12

**Authors:** Ali Nouri

**Affiliations:** Department of Animal Science, Garmsar Branch, Islamic Azad University, Garmsar, Iran

**Keywords:** Immunology, Microbiology

## Abstract

The study was conducted to consider the anticoccidial and immunogenic effectivities of encapsulated organic acids and anticoccidial drugs in broilers reared on a reused litter infected with *Eimeria* spp. for simulating in-field exposure to avian coccidiosis. 525 *mixed*-sex one-day-old broiler chicks (Ross 308) were used in a 2 × 3 factorial experiment as a completely randomized design with seven experimental groups and five replicates of 15 chicks. The seven experimental groups were included: negative (uninfected; T_1_) and positive (infected; T_2_) control groups fed a diet without additive, and other infected groups (T_3_–T_7_) fed diets supplemented with 0.05% maduramicin, 0.02% diclazuril, 0.1% EOAs, 0.05% maduramicin and 0.1% EOAs, 0.02% diclazuril and 0.1% EOAs. During the experimental period, the evaluated parameters were European production efficiency factor (EPEF; at 22 days of age (d)), oocyst output per gram feces (OPG; at different ages), oocyst reduction rate (ORR; at 22-d), survival rate (SR; at 22-d), caecal lesion score (CLS at 22-d), sporulation percentage (SP; by in vitro anticoccidial tests), bloody diarrhea (BD; by scoring the bloody feces each morning from 13 to 31-d), immunity (humoral test at 28 and 35-d and cell-mediated test at 22-d), goblet cells analysis of the jejunum (GC; at 22-d) and anti-coccidiosis index (ACI; at 22-d). EOAs and anticoccidials, especially their simultaneous feeding improved (*P* < 0.05) broiler’s EPEF, SR, OPG, ORR, SP, CLS, immunity and BD (scored). ACI was improved (*P* < 0.05) by EOAs more than anticoccidials (marked vs. moderate). The highest ACI was significantly observed in EOAs + diclazuril group. EOAs as a safe alternative had more intensive anticoccidial and immunogenic properties and increased the anticoccidial drugs’ effectiveness, especially diclazuril in *Eimeria* spp-infected broilers.

## Introduction

In the overall world, commercial broiler flocks are primarily floor raised in enclosed, environmentally controlled facilities. Within these commercial broiler houses, poultry litter can be reused for a year or longer if managed well and maintained in a relatively dry state. Reuse of poultry litter is a solution for managing and preventing the disposal of the large quantity of poultry litter produced (one of the greatest challenges) in the commercial poultry industry^1^. Although the reuse of poultry litter can help alleviate this challenge, there is concern that the reused litter environment, both biotic and abiotic, may negatively affect the intestinal microbiota of the bird, potentially resulting in intestinal infection^2^, poor health and reduced production efficiency^3,4^. Coccidiosis, which is caused by seven species of intracellular protozoan parasites of the *genus Eimeria*, is a rapidly developing intestinal disease that presents with bloody diarrhea and listlessness and can cause high levels of mortality in commercial poultry flocks especially reared on reused litter^3^. Jenkins et al. (2019) indicate that Eimeria oocysts survive in the house and litter and have the ability to infect newly hatched chicks^2^. *Eimeria* oocysts are highly infectious and resilient and by invading intestinal epithelium cells cause the destruction of the infected cells, resulting in reduced performance and increased morbidity and mortality^2^. The regular administration of coccidiostats in feed and preventive vaccinations are typical methods used to control the disease and improve^5^. Supplementing the diet with diclazuril had a positive effect on improving the impaired intestinal morphology^6^ and reducing mortality, oocyst shedding^6,7^ and the severity of lesion scores^8^ in *Eimeria*-infected groups. The effect of ionophorous anticoccidials (such as salinomycin or maduramicin) on the chicken coccidian is via disrupting ion gradients across the cell membrane of the Eimeria parasite^9^. Infected birds treated ionophore drugs have shown significant improvements in goblet cells number and signs of coccidiosis, including bloody diarrhea and oocyst suppression in feces^4,9^. However, there have been increasing concerns about the occurrence of drug-resistant oocytes and the drug residues in broilers' meats and also the production capacity of current vaccine lines is limited^10^. Thus, the global poultry industry is urged to develop alternatives to anticoccidials for sustainable antimicrobial-free poultry production. In response to these increasing global needs, nutrition-based strategies have been implemented to control avian coccidiosis caused by the *Eimeria* spp.^4^. Among the potential candidates as alternatives, organic acids (OAs) have been explored as they exhibit various biological properties including antimicrobials, modulation of intestinal pH, enrichment of the immune system, protection of pH-sensitive intestinal pathogen and changes in the villus height and depth in the small intestine^11^. By lowering the pH of the ceca, oocysts are negatively affected resulting in a less severe lesion score^12^.

Organic acids commonly used in animal husbandry include formic acid, acetic acid, propionic acid, butyric acid, lactic acid, benzoic acid, citric acid, malic acid, and fumaric acid. Among them, formic acid, acetic acid, butyric acid (having an unpleasant odor), and propionic acid are highly volatile and highly corrosive and when used in large amounts have an adverse effect on reducing feed intake due to their disagreeable flavor. In the case of salt-type organic acids, organic acids are eluted from the digestive organs due to the elimination of salts. In this case, the organic acids in the form of dissociated organic acids, which have lost their acidity, are hardly antimicrobial^11^.

With the development of coating technology, protected acids by encapsulation for targeted delivery to different gut segments have gained considerable attention. Studies’ results have indicated that encapsulated organic acids (EOAs) improved the growth performance of broilers^13,14^. This may be due to the fact that non-encapsulated additives are mainly metabolized and absorbed in the proximal part of the digestive tract and rarely reach its distal part^15^, while the EOAs can be slowly released along the intestine, especially distal ileum, caecum and colon, which results in improved intestine health and performance and interfering the survival rate of enteric pathogens^16,17^. Acidification with various OAs has been reported to reduce the production of toxic components by colonized pathogens on the intestinal wall, thus preventing the damage to epithelial cells and finally improving performance and gut health^18,19^. Therefore, the decrease of enteric pathogens especially protozoan parasites of the *genus Eimeria* in the intestine would be of great significance to the poultry industry.

The objective of this study was to evaluate the effects of dietary supplementation of EOAs and two selected anticoccidials (maduramicin and diclazuril) in the diet on European production efficiency factor (EPEF), oocyst output per gram feces (OPG), oocyst reduction rate (ORR), survival rate (SR), caecal lesion score (CLS), sporulation percentage (SP), bloody diarrhea (BD), cell-mediated and humoral immune responses, goblet cells (GC), anti-coccidiosis index (ACI) in broiler chickens reared on a reused litter infected with *Eimeria* spp. for simulating in-field exposure to avian coccidiosis.

## Materials and methods

### Experimental animals and design and feed preparation

The study was carried out utilizing 525 *mixed*-sex one-day-old industrial healthy broiler chicks (Ross 308) reared under experimental coccidiosis conditions (assuming the probability of the effect of supplementing EOAs and selected anticoccidials in the diet on the studied traits of infected chickens with *Eimeria* spp.). At 1 day of age, the healthy chicks were individually weighed, then selected for approximately the same weight (40 g) and randomly assigned into the seven groups having five replicates of 15 chicks (35 pens (1.0 × 1.8 × 0.75 m)), basis on a 2 × 3 factorial experiment as a completely randomized design (refer to the statistical analysis section). The chicks were reared on the litter system under standard management conditions in three experimental (starter, grower, and finisher) periods until 42 days of age. The ad libitum feeding program included a starter diet for 1–10 days, a grower diet for 11–25 days and a finisher diet for 26–42 days of age. The birds in the negative control group (T1) reared from the hatch on a sterilized litter. A diet without additives was given to the negative (NC; T1) and positive (PC; T2) control groups. The control diet was formulated according to the standards prescribed in Ross 308 Broiler Nutrition Specification, June 2007^20^. The ingredient and chemical composition of the control diet are listed in Table 1. In order to facilitate colonization of adult-type gut protozoan parasites under experimental conditions and simulate in-field exposure to avian coccidiosis^2,4^, chickens of other treatments (T2 to T7) were reared from the hatch on used litter from a poultry farm challenged with *Eimeria spp*.. The other five treatment groups were given the same diet as fed to the control groups but were supplemented with 0.05% maduramicin (T3), 0.02% diclazuril (T4), 0.1% encapsulated organic acids (EOAs; T5)^18^, 0.05% maduramicin and 0.1% EOAs (T6), 0.02% diclazuril and 0.1% EOAs (T7). The anticoccidials of diclazuril (Diclacid 0.5%) and maduramicin (Maduramicin 1%) and EOAs (comprised of 15% of lactic acid, 10% of acetic acid, 5% of benzoic acid, 5% of formic acid, and 5% of citric acid) were mixed thoroughly in aforesaid quantities to a small amount of feed (1 kg) in a premixer. The resultant mixture was then mixed with the rest of the feed in a mechanical blender until a thorough and consistent mixture was obtained.

**Table 1:** Ingredients and nutrient contents of basal diets in different experimental periods.

	Starter	Grower	Finisher
**Ingredients (g/kg)**			
Corn	570	604.10	647.60
Soybean meal	371.40	342.20	297
Soybean Oil	12.0	16	2
Calcium carbonate	12.60	10.50	10.20
Dicalcium phosphate	18	15.20	14.60
Common Salt	2.10	2	2
Sodium bicarbonate	1.60	1.70	1.70
DL-Methionine	2.90	2	1.40
HCl-Lysine	2.40	1	0.50
L-Threonine	1	0.30	0
Vitamin & Mineral Premix 1	6	5	5
**Nutrient contents**			
Metabolisable Energy (MJ/kg)	12.13	12.43	12.71
Crude Protein (g/kg)	210.90	198	180.50
Methionine (g/kg)	4.50	4	3.50
Methionine + Cysteine (g/kg)	9	7.90	6.90
Lysine (g/kg)	12.20	10.40	8.90
Threonine (g/kg)	8	6.9	6
Tryptophan (g/kg)	1.90	1.70	1.40
Arginine (g/kg)	12.60	10.70	9.40
Calcium (g/kg)	10.10	8.50	8.10
Available Phosphorus (g/kg)	4.80	4.20	4
Na (g/kg)	1.50	1.50	1.50
Linoleic Acid (g/kg)	12	11.30	9.50

^1^The premix is included A: 10,000 IU, D_3_: 5000, E: 50 IU, K: 3 mg, B_1_: 2 mg, B2: 6 mg, Niacin: 60 mg, Pantothenic Acid: 15 mg, B_6_: 3 mg, Biotin: 0.10 mg, Folic Acid: 1.75 mg, B_12_: 0.016; Cu: 16 mg, I: 1.26, Fe: 40 mg, Mn: 120 mg, Se: 0.30 mg, Zn: 100 mg.

### Characterization of encapsulated organic acids

#### Encapsulation method for producing EOAs

EOAs used in the current study were produced through a matrix coating technology in the University Research Centre and laboratory (the invention has not yet been registered and more details will be reported instantly after registering it). Active ingredients including lactic acid, acetic acid, benzoic acid, formic acid, citric acid (15, 10, 5, 5, and 5% respectively) are dispersed in a matrix of shell material, a mixture of lipids that can allow the active components to reach the intestine in an intact form, and be released slowly by the reaction of lipase from the intestine thereby showing beneficial effects to animals^16,21^. Finally, the prepared suspension was sprayed into a cooling chamber to prepare encapsulated organic acid particles.

### Encapsulated particle size

The encapsulated particles size was measured using a Nicomp submicron particle sizer (Model 370, Santa Barbara, California). Ten µ EOAs suspension was taken and suitably diluted to measure the mean diameter of the capsules.

### Encapsulation efficiency (% EE)

Encapsulation efficiency was determined as the percentage of OAs incorporated into lipid capsules (EOAs) relative to the initial amount of organic acids added. The encapsulated particles were lysed using 10% Triton X-100 to determine the amount of OAs present. Briefly, 25 µL of the suspension was added to 975 µL of 10% Triton X-100 and vortexed for 5 min to facilitate lysis of the capsules. The supernatant (100 µL) was taken and used for HPLC (high-performance liquid chromatography) analysis. Encapsulation efficiency was calculated using^22^:

$$Encapsulation \,efficiency \left( \% \right) = \frac{EOAs}{{IOAs}}*100$$

where EOAs is the amount of OA present in capsules after lysis with 10% Triton X-100 and IOAs is the amount of OA added initially.

### Used litter

Used litter was obtained from a commercial broiler house of a farm challenged with coccidiosis using diclazuril as a coccidiosis control program, where at least 10 broiler flocks had previously been grown. The litter was transported to the research facility for the current study, homogenized, and evenly distributed into pens of treatments 2 to 7. The presence of *Eimeria* oocysts in the used litter for all cited treatment groups was ~ 10^7^/g (± 1.80 SD) when enumerated using a hemocytometer. *Eimeria acervulina, Eimeria maxima, Eimeriamitis, Eimeria praecox, and Eimeria tenella*, but not *Eimeria brunetti* or *Eimeria necatrix*, were identified by PCR using coccidia species-specific primers^23^. An unused and sterilized litter was distributed into pens of treatment 1.

### European production efficiency factor (EPEF)

EPEF was calculated using the method suggested by^24^:

$$EPEF = \frac{{Livability*Live weight \left( {kg} \right)}}{Age in days*FCR}*100$$

Data on body weight and feed intake were recorded during experimental period and mortality was recorded at the occurrence. From the above data, the feed conversion ratio was calculated as hen day. The EPEF was calculated and evaluated for 22 days of age, because the highest challenge was occurred at this age. The EPEF was calculated in the infected broilers at 22 days of age because the highest challenges occurred at this age.

### Oocyst per gram (OPG) and caeca lesion score (CLS)

The flock was monitored for signs of disease and mortality. For the OPG of feces, the oocysts were counted by the McMaster technique as described earlier^25^. The calculation of OPG was performed at 4, 7, 10, 13, 19, 22, 25, 28, 31, 34, 37, 42 days of the experimental period and their average was reported.

To determine CLS, five chickens from each group were randomly chosen at 22 days of age [21 DPI (days post raising on infected litter)]. For sacrificing, chickens were stunned using CO2, then exsanguinated by cutting the jugular vein. Lesion scoring was performed according to the method was suggested by Song et al*.*^26^.

### Bloody diarrheal score (BDS)

The bloody diarrheal score is a qualitative estimation of the deviation of the fecal appearance from a normal state. It was obtained by scoring the bloody feces each morning from 13 to 31 days of age (12 to 30 DPI). The oocyst counts indicated the onset time of intensifying infection was 10–13 days of age. The BDS ranged from 0 to 4: A score of 0 indicated normal feces without hemorrhage; a score of 1 indicated 1–25% hemorrhage in the feces; a score of 2 indicated 25–50% hemorrhage in the feces; a score of 3 indicated 51–75% hemorrhage in the feces; and a score of 4 indicated 76–100% hemorrhage in the feces^27^.

### In vitro anticoccidial tests

Fresh fecal samples from infected birds were collected and mixed in 50 ml water. After vigorously shaking the tube, it was filtrated through a single thickness of muslin. The procedure to harvest oocysts was by floatation technique using saturated salt (NaCl) solution. To determine the sporulation time of the oocysts, the salt solution was removed by washing the oocysts with phosphate-buffered saline and centrifuged 3–4 times at 1,500 M for 2 min in graduated tubes. Finally, 10 ml of tap water was added to the salt-free oocyst suspension and oocyst per 1 ml was calculated. A number of 100 oocysts were added to dishes that contained the treatments and the set-up was left at ambient temperature and oxygen. The suspensions were examined by the hemocytometer chamber at hours 12, 24, 48, and 72 to determine the sporulation time of oocysts. Then, the percentages of sporulated oocysts were recorded^28^.

### Immune responses evaluation

Two in-vivo tests in which cutaneous response to phytohemagglutinin­P (PHA­P) and serum antibody level produced in response to sheep red blood cells (SRBCs) were measured to evaluate the immune competence of the experimental diets in the infected broilers.

### Cell-mediated immunity

Cell­mediated immunity was assessed through injection of PHA­P (Sigma­Aldrich, St. Louis, MO, USA) as reported earlier^29^ with the following slight modification. Briefly, two birds per replicate (n = 10 per group) were randomly selected at 22 days and the PHA­P solution (prepared in sterile phosphate­buffered saline [PBS]) was injected intradermally (100 μg/100 μL/chicken) between the 3rd and 4th digits of the right foot. The left foot served as control and was injected with 100 μL of PBS. The net increase in thickness of the injected sites was evaluated on 6, 12, and 24 h post­injection using pressure sensitive micro­ meter. The immune response (foot web index) to PHA­P was measured by subtracting the left foot thickness from that of the right foot.

### Antibody response to SRBCs (humoral immunity)

Two birds per treatment replicate (n = 10 per group) were randomly selected, wing banded and injected bilaterally with 5% SRBCs antigen (sheep (kept at the University Research Center) blood collected in Alsever’s solution, washed thrice and suspended in phosphate buffer saline) in two parts (0.5 mL each, intramuscularly in both sides of the Musculus pectoralis) at 14 days of age. A booster dose was given at 21 days of age. Blood samples were collected on seven days post-primary injection and at 35 days of age. Sera separated (2000 × g for 10 min) and were stored at − 20 °C till analysis. The antibody response to SRBCs was measured using hemagglutination assay (HA), as previously mentioned^30^.

### Goblet cells (GC) analysis of the jejunum

The GC were analyzed as previously described^31^ at 22 days of age. Briefly, two chickens per treatment replicate were randomly selected and sacrificed. ∼2-cm-long jejunal samples, taken midway between the endpoint of the duodenal loop and Meckel’s diverticulum, were collected, flushed with 10% neutral buffered formalin and fixed overnight in 10% neutral buffered formalin. Goblet cells were visualized by periodic acid–Schiff staining. The area of the GC in the jejunum was counted based on the length and width of the goblet cell “cup” in cross-sections of the villi under an Olympus light microscope (Olympus Optical Co., Beijing, China). The density of goblet cells was calculated as the number of goblet cells per unit surface area (mm2).

### Anti-coccidiosis index (ACI)

The ACI of each group was calculated as stated by Lan et al*.*^27^:

$$\begin{aligned} The ACI = & \left( {Relative\, weight \,gain \,rate + Survival \,rate} \right) \\ & \quad - \left( {Oocyst \,value + lesions \,score} \right) \\ \end{aligned}$$

$$Relative\, weight \,gain \,rate = \frac{Average \,weight \,gain \,in \,each \,group}{{Average \,weight \,gain \,in \,NC \,group}} \times 100$$

$$Survival \,Rate = \frac{Number \,of \,surviving \,chickens}{{Initial \,number \,of \,chickens}} \times 100$$

$$Oocyst \,value \left( \% \right) = \frac{Oocysts \left( g \right) output \,of \,every \,group}{{Oocysts \left( g \right)output \,of \,the \,PC \,group}} \times 100$$

$$Oocyst\, reduction \,rate \left( \% \right) = \frac{OPG \,of \,PC \,group - OPG \,of \,experimental \,group}{{OPG \,of \,PC \,group}} \times 100$$

At 1 and 22 days of age [0 and 21 DPI (days post raising on infected litter)], all the chickens in each replicate were weighed to get the starting and final BW, respectively. The BWG has been calculated as the final BW minus the initial BW. The highest challenges occurred in the infected broilers at 22 days of age; therefore, the ACI value was calculated at this age.

In brief, the ACI value below 120 was evaluated to be an inactive anticoccidial effect, 120 to 140 as mild or slight, 140 to 160 as moderate, 160 to 180 as marked, and above 180 as excellent.

### Blinding and statistical analysis

Before starting the experiment, the treatments were coded and randomly assigned to the experimental units (with similar weight and management conditions) based on the randomization table. During the experiment, the diets were fed to the birds based on coding treatments. Feeding diets, sampling, injections and measurements were performed by skilled individuals. The experimental data were sent as coded treatments and traits to a statistical expert for analysis.

Because of the NC group’s (T1) data was used only for calculation of ACI for treatments (T2–T7) fed to infected birds. Therefore, statistical analysis of the final results for all parameters was basis on data, measurements and calculations related to these treatments (T2–T7). The data obtained on various parameters (for T2–T7) were subjected to statistical analysis using the GLM (general linear model; method of variance analysis) procedure of SAS software Ver. 9.3^32^ based on a 2 × 3 factorial experiment as a completely randomized design (CRD) with five replications by the following model:

$$Y_{ijk} = \mu + a_{i} + \beta_{j} + a\beta_{ij} + e_{ijk}$$

$Y_{ijk}$ is evaluated trait or parameter; $\mu$ is the mean; $a_{i}$ is the EOAs; $\beta_{j }$ is the anticoccidial type; $a\beta_{ij}$ is the interaction of EOAs × anticoccidial type; $e_{ijk}$ is error.

For analysis of the data, the main sources of variance were EOAs, the anticoccidial type and the interaction among them (experimental treatments). After statistical analysis of factors and treatments effects, Duncan’s test was used to elucidate differences between the means, with the 0.05% level considered significant. Results are reported as the mean and pooled standard error of the mean (SEM).

### Ethics approval and consent to participate

The experiment was conducted in accordance with ARRIVE guideline^33^, also the guidelines set out in the joint publication of the National Health and Medical Research Council of Australia, CSIRO and the Australian Agricultural Council entitled ´Code of Practice for the Care and Use of Animals for Experimental Purposes´^34^. Also, institutional and national standards for the animals’ care and welfare and the appropriate measures to minimize pain, stress or discomfort were used. This study was approved by the ethics Committee (permit no. 1523/12. Date: 2021) of Department of Animal Science (Garmsar Branch, Islamic Azad University, Garmsar, Iran).

## Results

### Characterization of encapsulated OAs (EOAs)

The results obtained by Nicomp submicron particle sizer and HPLC indicated that the EOAs’ particle size and encapsulating efficiency (EE) were 105 ± 20 nm and 74%, respectively.

### European production efficiency factor (EPEF)

The effect of using EOAs and anticoccidials in the diet on EPEF of broiler chickens infected with *Eimeria* is represented in Table 2. Adding EOAs to the diet affected significantly (*P* < 0.05) EPEF at 22 days of age. Using EOAs in diet increased (*P* < 0.05) EPEF in the broiler chickens.

**Table 2:** Effect of EOAs and anticoccidials on EPEF^1^, SR^2^, CLS^3^, ORR^4^ and ACI^5^ in infected broilers.

Factor	EPEF1	SR2	CLS3	ORR4	ACI5
**EOAs:**					
No Added	254^b^	87.67^b^	3.39 a	42.90^b^	115^b^
Added	306^a^	95.33^a^	2.76^b^	83.70^a^	168^a^
SEM^6^	2	0.64	0.043	0.41	0.77
*P* value	0.01	0.02	0.03	0.04	0.03
**Anticoccidials:**					
No Added	257^c^	86.62^c^	3.50^a^	40.7^b^	112^c^
Maduramicin	285^b^	92.37^b^	2.97^b^	74.2^a^	154^b^
Diclazuril	298^a^	95.50^a^	2.75^c^	75.5^a^	158^a^
SEM	2.4	0.78	0.05	0.51	0.94
*P* value	0.03	0.02	0.03	0.04	0.04
**EOAs × Anticoccidials:**					
No additive (T2)	229^e^	80.00^d^	3.95^a^	0.00^d^	63 f.
Maduramycin (T3)	262^d^	90.00^c^	3.14^b^	63.7^c^	139^e^
Diclazuril (T4)	271^d^	93.00^bc^	3.08^b^	65.0^c^	143^d^
EOAs (T5)	284^c^	93.25^bc^	3.05^b^	81.4^b^	161^c^
EOAs + Maduramycin (T6)	307^b^	94.75^ab^	2.80^c^	84.6^a^	169^b^
EOAs + Diclazuril (T7)	325^a^	98.00^a^	2.43^d^	85.0^a^	174^a^
SEM	3.4	1.1	0.07	0.72	1.33
*P* value	0.04	0	0.01	0.04	0.02

^a–b^ In each column, means with no same (a, b) superscript differ significantly in *p < 0.05* value.

^1^ European production efficiency factor; ^2^ Survival rate; ^3^ Caecal lesion score, ^4^ Oocyst reduction rate; and ^5^ Anti-coccidiosis index have been calculated in broilers at 22 days of age.

^6^Pooled standard error of mean.

The results of Table 2 indicate that the effect of using dietary anticoccidials was significant (*P* < 0.05) on EPEF of infected broilers. Feeding the diets containing anticoccidials to the broilers increased (*P* < 0.05) their EPEF than those fed diets without anticoccidial. This effect was significantly more for diclazuril.

The simultaneous effect of used EOAs and anticoccidials was significant (*P* < 0.05) on infected broilers’ EPEF (Table 2). Although diet containing EOAs increased significantly broilers’ EPEF compared to the positive control and anticoccidials groups; but, the most significant increase was achieved by adding EOAs in diets containing anticoccidials especially diclazuril.

### Oocyst reduction rate (ORR) and output per gram feces (OPG)

Effect of feeding dietary EOAs and anticoccidials on oocyst reduction rate (ORR; at 22 days of age) and oocyst output per gram feces (OPG; at different ages) in infected broilers is represented in Tables 2 and 3, respectively. Evaluating these results indicated that the use of EOAs in the diet decreased (*P* < 0.05) significantly OPG (at 10 until 42 days of age) and ORR in the broilers.

**Table 3:** Effect of EOAs and anticoccidials on OPG^1^ in broilers infected with *Eimeria* (× 1000).

Days													
Factor	4	7	10	13	16	19	22	25	28	31	34	37	42
**EOAs:**													
No Added	0.48	1.17	2.65^a^	3.62^a^	4.49^a^	6.24^a^	13.94^a^	8.44^a^	4.61	2.68 a	1.67^a^	1.10^a^	0.54^a^
Added	0.44	1.21	1.55^b^	1.46^b^	1.82^b^	2.52^b^	3.99^b^	1.77^b^	0.96^b^	0.56^b^	0.52^b^	0.34^b^	0.21^b^
SEM^2^	0.04	0.03	0.03	0.06	0.07	0.09	0.11	0.06	0.03	0.02	0.01	0.01	0.01
*P* value	0.48	0.34	0.01	0.02	0.03	0.04	0.02	0.03	0.02	0.04	0.03	0.03	0.04
**Anticoccidials:**													
No Added	0.49	1.23	3.04^a^	3.99^a^	4.96^a^	6.88^a^	14.48^a^	8.42^a^	4.59^a^	2.68^a^	1.65^a^	1.07^a^	0.53^a^
Maduramicin	0.48	1.16	1.61^b^	1.83^b^	2.27^b^	3.16^b^	6.31^b^	3.53^b^	1.93^b^	1.12^b^	0.82^b^	0.55^b^	0.30^b^
Diclazuril	0.4	1.19	1.63^b^	1.80^b^	2.24^b^	3.11^b^	6.10^b^	3.37^b^	1.84^b^	1.07^b^	0.81^b^	0.54^b^	0.29^b^
SEM	0.06	0.04	0.04	0.07	0.87	0.12	0.13	0.07	0.04	0.02	0.01	0.01	0.01
*P* value	0.48	0.47	0.02	0.04	0.03	0.03	0.04	0.03	0.04	0.02	0.03	0.01	0.02
**EOAs × Anticoccidials:**													
No additive (T2)	0.54	1.16	4.47^a^	6.54^a^	8.12^a^	11.27^a^	24.40^a^	14.48^a^	7.90^a^	4.61^a^	2.64^a^	1.74^a^	0.81^a^
Maduramycin (T3)	0.49	1.14	1.71^b^	2.21^b^	2.74^b^	3.81^b^	8.86^b^	5.51^b^	3.00^b^	1.75^b^	1.19^b^	0.79^b^	0.41^b^
Diclazuril (T4)	0.41	1.23	1.76^b^	2.11^b^	2.62^b^	3.64^b^	8.55^b^	5.35^b^	2.92^b^	1.70^b^	1.18^b^	0.78^b^	0.41^b^
EOAs (T5)	0.45	1.29	1.61^bc^	1.44^c^	1.79^c^	2.49^c^	4.55^c^	2.36^c^	1.29^c^	0.75^c^	0.66^c^	0.41^c^	0.26^c^
EOAs + Maduramycin (T6)	0.46	1.19	1.52^c^	1.45^c^	1.81^c^	2.51^c^	3.76^d^	1.55^d^	0.85^d^	0.49^d^	0.46^d^	0.32^d^	0.19^d^
EOAs + Diclazuril (T7)	0.4	1.16	1.51^c^	1.49^c^	1.85^c^	2.58^c^	3.65^d^	1.39^d^	0.76^d^	0.44^d^	0.45^d^	0.31^d^	0.18^d^
SEM	0.08	0.05	0.05	0.09	0.12	0.17	0.19	0.1	0.057	0.03	0.02	0.01	0.01
*P* value	0.85	0.17	0.016	0.02	0.01	0.03	0.04	0.02	0.04	0.04	0.03	0.01	0.02

^a-^b In each column, means with no same (a, b) superscript differ significantly in *p < 0.05* value.

^1^OPG: Oocyst output per gram feces; ^2^Pooled standard error of mean.

The effect of adding anticoccidials to diet was (*P* < 0.05) significant on decreasing infected broilers’ OPG (at different ages of 10 days) and ORR. The effect was significantly similar for diets containing maduramicin or diclazuril.

The interaction effect of EOAs and anticoccidials added to diet was significant (*P* < 0.05) on decreasing OPG (during 10–42 days of age) and ORR in infected broiler chickens. The highest effect on OPG and ORR was achieved (*P* < 0.05) by feeding diets containing EOAs and anticoccidials to the broilers at the ages.

### Survival rate (SR)

The effect of EOAs and anticoccidials added to the diet on SR of infected broiler chickens during the period of 1–22 days is represented in Table 2. Using EOAs in the diet increased significantly (*P* < 0.05) SR in the broilers.

Also, the effect of using dietary anticoccidials was significant (*P* < 0.05) on increasing infected broilers’ SR. The feeding a diet containing diclazuril caused (*P* < 0.05) the highest SR in the broilers.

The interaction effect of EOAs and anticoccidials was significant (*P* > 0.05) on improving SR in infected broilers. The simultaneous usage of EOAs and anticoccidials in diet had (*P* > 0.05) more significant effect on broilers’ SR. The highest SR was significantly obtained by feeding diet containing diclazuril and EOAs to the broilers.

### Caecal lesion score (CLS)

The CLS in infected broiler chickens fed diets containing EOAs and anticoccidials are shown in Table 2. Adding EOAs to diet decreased significantly (*P* < 0.05) the broilers’ CLS.

With regard to Table 2, the effect of using dietary anticoccidials was significant (*P* < 0.05) on decreasing values of CLS in infected broilers. The broilers fed dietary diclazuril showed significantly the lowest CLS as compared to other groups.

Also, the results of Table 2 represent (*P* < 0.05) the significant interaction effect of EOAs and anticoccidials on CLS in infected broilers. Feeding diets containing EOAs and anticoccidials especially diclazuril had more effect on decreasing CLS as compared to feeding diets containing each EOAs or anticoccidials to the broilers.

### In vitro anticoccidial effects

Table 4 represents in vitro anticoccidial effect of EOAs and anticoccidials on the sporulation percentage (SP) of *Eimeria* oocysts in different hours of in vitro assay. The EOAs decreased (*P* < 0.05) SP of *Eimeria* oocysts during the assay period (from 12 until 72 h after beginning the assay).

**Table 4:** In vitro anticoccidial effect of EOAs and anticoccidials on the SP^1^ in infected broilers.

Factor	Number of Hours			
	12	24	48	72
**EOAs:**				
No Added	68.25^a^	77.83^a^	84.92^a^	87.25^a^
Added	46.33^b^	63.17^b^	65.83^b^	71.33^b^
SEM 2	1.04	1.02	0.76	0.6
*P* value	0.01	0.03	0.04	0.04
**Anticoccidials:**				
No Added	65.63^a^	75.88^a^	82.50^a^	85.50^a^
Maduramicin	58.13^b^	69.87^b^	74.75^b^	79.37^b^
Diclazuril	48.13^c^	65.75^c^	68.88^c^	73.00^c^
SEM	1.27	1.25	0.93	0.73
*P* value	0.02	0.03	0.01	0.04
**EOAs × Anticoccidials:**				
No additive (T2)	74.5	85	95.00^a^	96.00^a^
Maduramycin (T3)	69.75	76.75	83.75^b^	87.50^b^
Diclazuril (T4)	60.5	71.75	76.00^c^	78.25^c^
EOAs (T5)	56.75	66.75	70.00^d^	75.00^d^
EOAs + Maduramycin (T6)	46.5	63	65.75^e^	71.25^e^
EOAs + Diclazuril (T7)	35.75	59.75	61.75^f^	67.75^f^
SEM	1.79	1.77	1.31	1.04
*P* value	0.15	0.22	0.02	0.04

^a–b^ In each column, means with no same (a, b) superscript differ significantly in *p < 0.05* value.

^1^ SP: Sporulation percentage of *Eimeria* oocysts.

^2^ Pooled standard error of mean.

The use of anticoccidials had a significant (*P* < 0.05) effect on decreasing the SP during different hours of the assay. Diclazuril showed the highest effect on decreasing the SP percentage.

With respect to the interaction effect of applying EOAs and anticoccidials on decreasing the SP, the results were (*P* < 0.05) significant from 36 until 72 h after beginning the assay. The most effect on SP was obtained by simultaneous usage of diclazuril and EOAs.

### Bloody diarrhea (BD)

**Figure 1:**
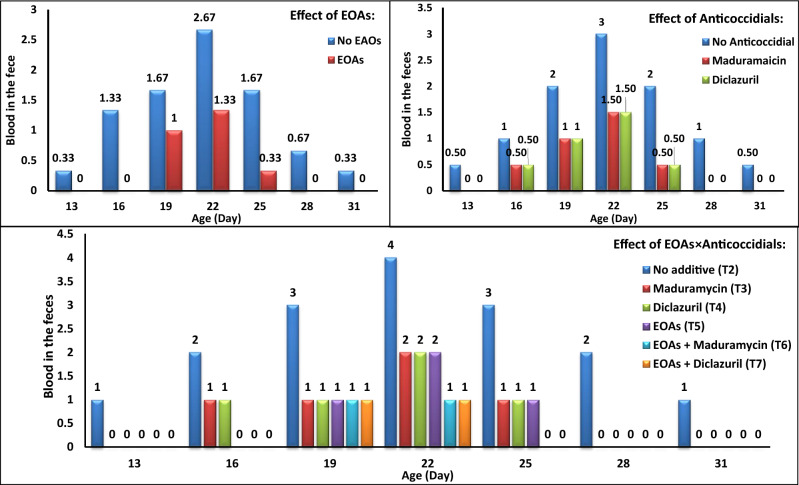
Effect of EOAs and anticoccidials on blood in the feces of broilers infected with *Eimeria.*

The effect of EOAs and anticoccidials used in the diet on blood in the feces of infected broilers is represented in Fig. 1. Using EOAs in the diet decreased bloody diarrhea in the broilers during 16–28 days of age. Also, feeding diets containing maduramicin or diclazuril decreased similarly BD during 13–31 days of age.

With respect to the simultaneous usage of EOAs and anticoccidials, BD was observed only in the positive control (no additive) group from 13 days of age. The extent of blood in feces was maximized until 22 days of age. Also, the BD occurred in birds fed by diets containing maduramicin or diclazuril without EOAs at 16 days of age. At 19 days, BD was observed in all of the infected experimental groups. The least extent of blood in feces was seen when feeding EOAs with maduramicin or diclazuril in the overall experimental period, especially at 22 days of age. Feeding diets containing EOAs with maduramicin or diclazuril caused the earliest time (25 days of age) of finishing the BD in broilers.

### Immune responses

#### Cell-mediated immunity (against phytohemagglutinin-P (PHA-P))

Table 5 represents the effects of dietary EOAs and anticoccidials on broilers’ immune response at different hours post PHA­P injection. The EOAs increased (*P* < 0.05) the immune response at 6, 12 and 24 h post PHA­P injection.

**Table 5:** Effect of dietary EOAs and anticoccidials on immune responses against PHA-P^1^ and SRBCs^2^ in infected broilers.

Factor	Time interval post injection PHA-P (h)			SRBCs	
	6	12	24	21	35
**EOAs:**					
No Added	0.57^b^	0.78^b^	0.98^b^	5.56^b^	7.15^b^
Added	0.61^a^	0.83^a^	1.05^a^	6.04^a^	7.87^a^
SEM2	0	0	0	0.04	0.05
*P* value	0.02	0.02	0.03	0.02	0.03
**Anticoccidials:**					
No Added	0.59	0.78^c^	0.98^c^	5.50^c^	7.10^c^
Maduramicin	0.58	0.82^b^	1.03^b^	5.86^b^	7.54^b^
Diclazuril	0.59	0.83^a^	1.04^a^	6.05^a^	7.88^a^
SEM	0	0	0	0.04	0.06
*P* value	0.948	0.03	0.043	0.04	0.01
**EOAs × Anticoccidials:**					
No additive (T2)	0.56	0.76^e^	0.95^e^	5.07^d^	6.51^d^
Maduramycin (T3)	0.56	0.79^d^	0.99^d^	5.71^c^	7.33^c^
Diclazuril (T4)	0.57	0.80^c^	1.00^c^	5.90^bc^	7.62^b^
EOAs (T5)	0.61	0.80^c^	1.01^c^	5.91^bc^	7.69^b^
EOAs + Maduramycin (T6)	0.61	0.84^b^	1.07^b^	6.01^ab^	7.76^b^
EOAs + Diclazuril (T7)	0.61	0.86^a^	1.08^a^	6.21^a^	8.14^a^
SEM	0.01	0.02	0.01	0.07	0.09
*P* value	0.95	0.02	0.03	0.04	0.03

^1^ Phytohemagglutinin-P.

^2^ Sheep red blood cells.

^a–b^ In each column, means with no same (a, b) superscript differ significantly in *p < 0.05* value.

^2^ Pooled standard error of mean.

There was no significant (*P* > 0.05) effect of using dietary anticoccidials on the immune response after 6 h post PHA­P injection. The used anticoccidials especially diclazuril increased (*P* < 0.05) the response at 12 and 24 h post PHA­P injection to broilers.

Although, the dietary simultaneous usage of dietary EOAs and anticoccidials had no effect on the response after 6 h post PHA­P injection to broilers. However, the effect was significant (*P* < 0.05) after 12 and 24 h post PHA­P injection. Adding EOAs to the diets containing anticoccidials especially diclazuril caused the most significant effect on the response.

### Humoral immunity (against sheep red blood cells (SRBCs))

The effects of dietary EOAs and anticoccidials on broilers’ antibody response to SRBCs at 21 and 35 days of age were represented in Table 5. The dietary EOAs increased (*P* < 0.05) the broilers’ response at 21 and 35 days of age.

The effect of feeding dietary anticoccidials to the broilers was significant (*P* < 0.05) on their antibody response to SRBCs at 21 and 35 days of age. The used anticoccidials especially diclazuril increased (*P* < 0.05) the response in the broilers at the cited ages.

The dietary simultaneous usage of EOAs and anticoccidials affected significantly (*P* < 0.05) antibody response to SRBCs in broilers at 21 and 35 days of age. At the cited ages, applying EOAs in the diets containing anticoccidials intensified the significant responses in the broilers. However, at 35 days of age, the most response was happened in the broilers fed a diet containing EOAs + diclazuril.

### Goblet cells (GC)

The effects of dietary EOAs and anticoccidials on jejunal goblet cells (GC) number at 10, 25 and 42 days of age in the infected broilers were represented in Table 6. At all cited ages, the EOAs increased significantly (*P* < 0.05) jejunal GC number in the infected broilers.

**Table 6:** Effect of EOAs and anticoccidials on jejunal GC^1^ number of infected broilers in different ages.

Factor	10 d	25 d	42 d
**EOAs:**			
No Added	21.09^b^	23.34^b^	25.45^b^
Added	22.65^a^	25.23^a^	26.75^a^
SEM^2^	0.1	0.08	0.05
*P* value	0.04	0.01	0.02
**Anticoccidials:**			
No Added	21.87	23.52^b^	24.99^b^
Maduramicin	21.83	24.61^a^	26.60^a^
Diclazuril	21.96	24.74^a^	26.71^a^
SEM	0.13	0.09	0.06
*P* value	0.84	0.04	0.03
**EOAs × Anticoccidials:**			
No additive (T2)	21.07	22.36^d^	24.51^d^
Maduramycin (T3)	21.05	23.73^b^	25.83^b^
Diclazuril (T4)	21.15	23.95^b^	26.01^b^
EOAs (T5)	22.66	24.68^c^	25.47^c^
EOAs + Maduramycin (T6)	22.61	25.49^a^	27.37^a^
EOAs + Diclazuril (T7)	22.69	25.53^a^	27.42^a^
SEM	0.18	0.13	0.08
*P* value	0.79	0.04	0.03

^a–b^ In each column, means with no same (a, b) superscript differ significantly in *p < 0.05* value.

^1^ GC: goblet cells.

^2^ Pooled standard error of mean.

The effect of anticoccidials independently or interacted with EOAs on increasing broilers’ jejunal GC number was insignificant (*P* > 0.05) at 10 days and significant (*P* < 0.05) at 25 and 42 days of age. However, adding EOAs to the diets containing anticoccidials had a more significant effect on increasing GC at the ages.

### Anti-coccidiosis index (ACI)

The effect of dietary EOAs and anticoccidials on ACI of infected broiler chickens at ages of 22 is shown in Table 2. EOAs used in the diet had a significant (*P* < 0.05) marked effect on ACI in broiler chickens at 22 days.

Also, applying dietary anticoccidials had a significant (*P* < 0.05) moderate effect on increasing ACI at 22 days. The feeding diet containing diclazuril caused (*P* < 0.05) the highest significant ACI in broilers.

The interaction effect of EOAs and anticoccidials was significant (*P* > 0.05) on improving the ACI at 22 days. At all ages, the effects were mild and moderate for diets containing maduramicin and diclazuril without EOAs, respectively. The significant effect of diet containing EOAs without anticoccidial was marked at 22 days. The simultaneous usage of EOAs and anticoccidials in diet had (*P* > 0.05) a more significant marked effect on broilers’ ACI. The highest ACI was significantly obtained by feeding a diet containing diclazuril and EOAs to broiler chickens at 22 days.

## Discussion

As expected, European production efficiency factor **(**EPEF) in the infected broilers untreated with EOA or synthetic anticoccidial drugs was most adversely affected which can be due to poor nutrient absorption and reduced immune responses resulting in intestinal tissue damage^9^. Using EOAs in the diet increased EPEF in broiler chickens. Indeed, the improved EPEF shown in this work could be due to growth-promoting effects of EOAs that enhanced the feed intake and digestibility^35^. Dietary EOAs in broilers improved EPEF related parameters^19,26^ due to improvement of digestive enzymes’ activity, pancreatic secretion and gut microbiota and morphology in terms of villus height and crypt depth in the small intestine^17,36^. Also with respect to our results, anticoccidials especially diclazuril added to the diet increased broilers’ EPEF. Diclazuril is a highly and more efficacious anticoccidial drug for improving performance and gut health^6^. The effect of ionophorous anticoccidials on the chicken coccidian is via disrupting ion gradients across the cell membrane of *Eimeria* parasite^9^.

The adding EOAs to the diets containing anticoccidials especially diclazuril caused more effect on increasing EPEF in broiler chickens. A reduction in the intracellular pH via the entry of undissociated acids into the coccidian cells and subsequent dissociation in the cytoplasm is the primary mode of organic acids action^37^ and this effect combined with the action mode of chemical anticoccidials have been caused the more interaction effect on EPEF.

The spread of oocysts in the feces is a risk factor for the prevalence of coccidiosis in intensive farming^8^. Results indicated that *Eimeria* infection increased the oocysts shedding in infected broilers during increasing age. On the contrary, the using EOAs or anticoccidials especially their interaction effect significantly reduced the oocyst output per gram feces (OPG) in broilers at different ages, suggesting that administrating these compounds especially their simultaneous usage in diet can play a significant role in controlling large scale avian coccidiosis epidemics in poultry farms. Wang et al*.* (2021) reported that dietary supplementation of *Lactobacillus plantarum* or diclazuril decreased the oocysts shedding^6^. In a study^35^, drinking acetic acid resulted in protective effects against *E. tenella* probably by declining pH of ceca and obliteration of oocysts in chickens. Supplementation of organic acids significantly reduced the oocyst index in broiler chickens. Acetic acid has been found to have anticoccidial properties against *E. tenella* in broiler chickens. By lowering the pH of the ceca, oocysts are negatively affected resulting in a less severe lesion score^8^. Dietary encapsulated essential oils and organic acids mixture improves gut health in broiler chickens challenged with necrotic enteritis^19^. Infected birds treated with salinomycin (as an ionophore drug) have shown significantly lower signs of coccidiosis, including bloody diarrhea and oocyst suppression in feces^9^. Clopidol and Diclazuril used in diet were effective in reducing oocyst shedding in broiler chickens^9^.

We found that using EOAs and anticoccidials (especially diclazuril) in the diet increase significantly Survival rate (SR) in broiler chickens. This significant effect was intensified with their simultaneous usage in the diet. Infective sporozoites enter the caecal mucosa by penetrating villus epithelial cells, resulting in extensive destruction of the caecal epithelium, occurrence of hemorrhagic feces and increase of eventually mortality^38,39^. Wang et al*.*, (2021) reported that dietary *Lactobacillus plantarum* supplementation inhibits oocyst shedding and improves the health of broilers infected with *Eimeria*, which was closely related to intestinal health and the regulation of gut microbiota and pH^6^. Dietary supplementation of *Lactobacillus plantarum* or diclazuril decreased the mortality and oocysts shedding in *Eimeria* treated broilers. On the contrary, it’s reported that the usage of blends of acetic, butyric, and lactic acids in diet did not reduce mortality in broiler chickens^40^.

Our results indicated that the Caecal Lesion score **(**CLS) in broiler chickens was decreased by feeding diets containing EOAs or anticoccidials, especially their simultaneous usage. Infected birds fed the EOAs-supplemented diet exhibited a higher villus height and villus height/crypt depth ratio, and reduced gut lesion^19^. Rao et al*.,* (2011) reported that usage of acetic acid (particularly 3%) in drinking water could overcome the intestinal lesions caused by the *Eimeria* species and subsequent malabsorption of nutrients^35^. Usage of dietary acetic acids and benzoic acids in feeding broiler chickens challenged with different species of *Eimeria* have been demonstrated a reduction in the severity of lesion scores^8,12^. Acetic acid has been found to have anticoccidial properties against *E. tenella* via lowering the pH of the ceca and negatively affecting oocysts resulting in a less lesion score in broiler chickens^8^. Gao et al*.* (2019) reported that supplementation of organic acids significantly reduced lesion scores compared to infected non-supplemented group^41^. Dietary encapsulated essential oils and organic acids mixture improves gut health in broiler chickens challenged with necrotic enteritis^19^. In a study^8^, supplementing the diet with diclazuril had a positive effect on reducing the severity of lesion scores in infected groups. The impaired intestinal morphology in the *Eimeria* infected broilers was also improved by Lactobacillus plantarum or diclazuril treatments^6^. Infected birds treated with salinomycin have shown significantly lower signs of coccidiosis, including bloody diarrhea^9^.

The EOAs or anticoccidials especially their simultaneous usage decreased significantly sporulation percentage (SP) during the in vitro assay period. Several in vitro experiments have shown beneficial effects when organic acids have been used individually or as a blend in chickens^14,28^. In vitro studies have demonstrated that an encapsulated blend of 4% thyme, 4% carvacrol, 0.5% hexanoic acid, 3.5% benzoic acid and 0.5% butyric acid retains its antimicrobial activity. The dietary administration of butyric acid induced positive effect on in vitro SP and chicken’s anticoccidial^14^. Balta et al. (2021) showed the anti-*Eimeria* efficacy of a natural antimicrobial mixture containing maltodextrin, sodium chloride, citric acid, sodium citrate, silica, malic acid, citrus extract, and olive extract on the infected broilers via in vitro test^28^.

The results of our study indicated the dietary usage of EOAs or anticoccidials decrease Bloody diarrhea (BD) in broiler chickens during 16–28 and 13–31 days of age, respectively. The effect was intensified by the simultaneous use of EOAs and anticoccidials in the diet. Feeding these diets caused the earliest time (25 days of age) of finishing the BD in broilers. Coccidiosis causes terrible BD in birds by destroying caecal and intestinal epithelial cells after entering infective sporozoites to the mucosa. In a study^35^, drinking of acetic acid resulted in protective effects against *E. tenella* probably by decreasing pH of ceca, obliteration of oocysts, and protection of the epithelium and finally decrease of BD in chickens. However, diclazuril were effective in reducing oocyst shedding^6^. The reduced bleeding can protect infected birds against secondary infections, inflammatory response, and toxic substances absorption^42^. Infected birds treated with salinomycin have shown significantly lower signs of coccidiosis, including such BD and oocyst suppression in feces^9^.

The obtained results demonstrated that feeding EOAs or anticoccidials (especially diclazuril) to broilers increase significantly their immune response against PHA­P or SRBC injections at the evaluated ages. The most significant effect on immune responses was achieved by supplementing EOAs in diets containing anticoccidials (especially diclazuril). The cell mediated immune response might be due to a delayed type of hypersensitivity. SRBCs act as Thymus dependent immunogens and are used for antibody response evaluation in chickens^43^. This indicates that EOAs especially short-chain organic acids may modulate the function of B and T cells in later stages of the antigenic exposure and can regulate the host immunity^44^. Park et al. (2015) noted that short-chain organic acids are commonly synthesized in the gut which supports the regulation and growth of Th1 and Th17 effector cells as well as interleukin­10 (IL­10) regulatory T­cells^45^. These cells maintain the immune system framework. Zhou et al. (2014) reported the effect of short-chain organic acids on the macrophage cell line^46^. They noted that the organic acids inhibited nitric oxide production, and diminished the cytokines expression, including IL­6, IL­10, interferon-gamma, and IL­1β, which controlled inflammation and maintained immune homeostasis. The organic acids regulate the macrophage activities in the intestine, and the macrophage effectuates the function of T cells and dendritic cells in the gut. The latter cells also have a role in host immunity. T-cell-mediated immune responses reduce the excretion of oocysts in animals infected with Eimeria and mainly involve CD4^+^ and CD8^+^ lymphocytes^47^. Therefore, the EOAs containing short-chain organic acids activated the immunomodulatory property through the production of host defense peptides^48^ and has no effect on provoking inflammation^49^.

**Figure 2:**
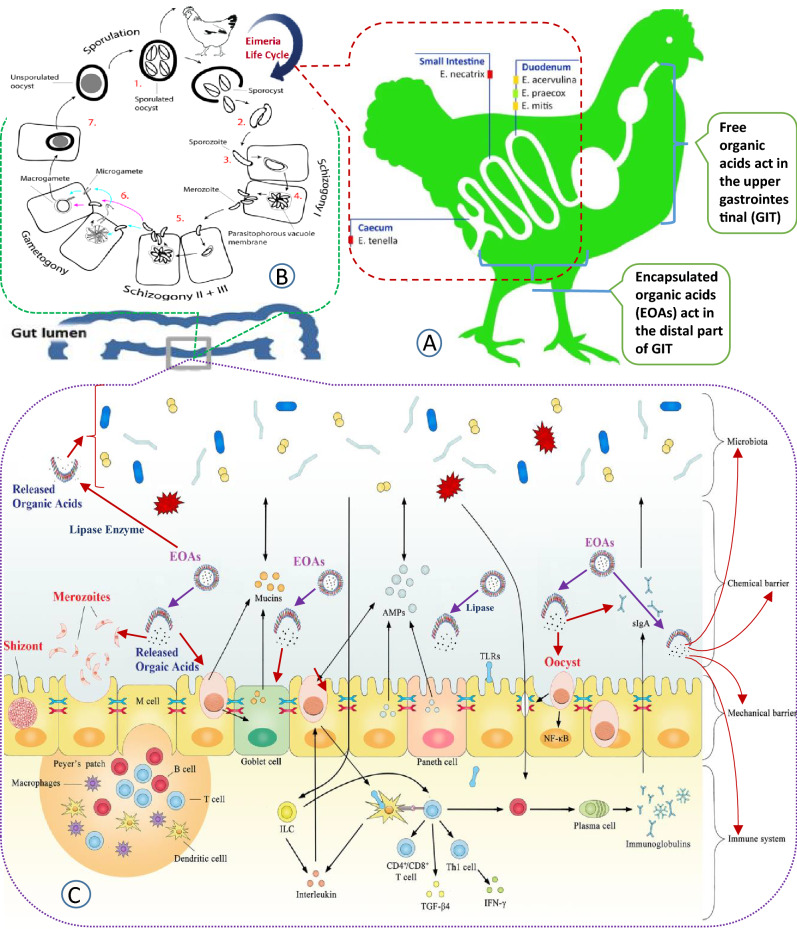
Summary of activity portions, mechanisms and effects of EOAs in the infected broilers’ gastrointestinal tract.

Legend: Part A. shows the activity portions of different forms of organic acids (free and encapsulated) as well as studied species of Eimeria in the gastrointestinal tract. The life cycle of Eimeria spp. has been shown in part B. Free organic acids are mainly metabolized and absorbed in the proximal part of the digestive tract and rarely reach its distal part, while the encapsulated organic acids (EOAs) can be slowly released along the intestine, especially the distal ileum, caecum and colon, which results in reducing growth and action of enteric pathogens especially protozoan parasites of the *genus Eimeria* and production of their toxic components, preventing the damage to epithelial cells, improving intestinal barrier structure (immune system, the mechanical barrier, chemical barrier, and gut microbiota) and performance of infected broilers (part C.).

Goblet cells (GC) via secreting Mucin covers the intestinal epithelial surface and plays a major role in protecting the intestinal epithelium from infection and pathogens and maintaining intestinal mucosal barrier integrity, immune hemostasis and gut health in infected broilers^50^. In the present study, increased GC number was observed in the birds administered by EOAs and anticoccidials especially their simultaneous usage, indicating that the addition helps more protect the gut barrier from direct contact with pathogenic bacteria in *Eimeria*-infected broilers. It is assumed that organic acids may uphold the GCs' activities by regulating their mucin gene and may positively contribute to the protective mechanisms in the gut^45^. The impaired intestinal morphology in the *Eimeria*-infected broilers was also improved by diclazuril treatment^6^. Infected birds treated with salinomycin as an ionophore drug have shown a significant improvement in goblet cells number^9^. Collectively, summary of our results and documented mechanisms of EOAs on decreasing *Eimeria* infection and lesions and improving immune system and the intestinal barrier structure have been represented in Fig. 2.

Our results indicated that pretreatment using of EOAs especially in diets containing anticoccidials could reduce the progress and development of intestinal inflammation, improve gut health and protect the intestinal barrier structure as evidenced by improving the gut lesions and bloody diarrhea in the infected broilers. The anti-inflammatory effect of EOAs has generally been attributed to their antimicrobial and immune-regulating actions^9^. The reduced intestinal inflammation may eventually lead to improved gut health and EPEF especially in chickens treated with diet containing EOAs and anticoccidials.

Our results indicated that the effect of dietary EOAs and anticoccidial on the anti-coccidiosis index (ACI) was significantly marked and moderate in the infected broilers, respectively. This significant effect was positively intensified with their simultaneous usage, especially with feeding diet containing EOAs and diclazuril to the broilers. Muthamilselvan et al. (2016) reported that chickens fed with a diet containing an organic acid had more significant higher ACI compared with the group treated with anticoccidial drugs^9^.

The beneficent results of simultaneous using EOAs and anticoccidials on the evaluated traits may be attributed to the fact that EOAs and anticoccidials are two active compounds in preparing an inappropriate environmental condition for the growth of many pathogenic and decreasing the intestinal colonization and infectious processes, ultimately decreasing the inflammatory reactions at the intestinal mucosa and barrier structure, which probably increase the gut health and functions of secretion, digestion, and absorption of nutrients by the mucosa based on cited studies.

## Conclusion

Compared to drug anticoccidials, EOAs as a safe and organic alternative had more effects on improving EPEF, OPG, ORR, SR, CLS, SP, BD, immunological response, GC and ACI in broiler chickens. Moreover, an interaction effect between these two substances was noticed on the evaluated traits. Therefore, it is beneficial and applicable to utilize EOAs, especially in diets containing anticoccidials in order to more improvement of the cited parameters in the broilers infected with *Eimeria* spp*.*

## Data Availability

The required information and materials and methods are reported in the current manuscript. If any additional data is required for the current study, it is available from the corresponding author on reasonable request.

## Abbreviations

ACI: Anti-coccidiosis index
BD: Bloody diarrhea
CLS: Caecal lesion score
DPI: Days post raising on infected litter
EOAs: Encapsulated organic acids
EPEF: European production efficiency factor
IR against PHA-P: Immunological response against Phytohemagglutinin-P
GC: Goblet cells
OAs: Organic acids
OPG: Oocyst output per gram feces
ORR: Oocyst reduction rate
SP: Sporulation percentage
SR: Survival rate
